# Integration of single-cell RNA-seq data into population models to characterize cancer metabolism

**DOI:** 10.1371/journal.pcbi.1006733

**Published:** 2019-02-28

**Authors:** Chiara Damiani, Davide Maspero, Marzia Di Filippo, Riccardo Colombo, Dario Pescini, Alex Graudenzi, Hans Victor Westerhoff, Lilia Alberghina, Marco Vanoni, Giancarlo Mauri

**Affiliations:** 1 Dept. of Informatics, Systems and Communication, University of Milan-Bicocca, 20126, Milan, Italy; 2 SYSBIO Centre of Systems Biology, 20126, Milan, Italy; 3 Dept. of Biotechnology and Biosciences, University of Milan-Bicocca, 20126, Milan, Italy; 4 Department of Research, Fondazione IRCCS Istituto Nazionale dei Tumori, Milan, Italy; 5 Dept. of Statistics and Quantitative Methods, University of Milan-Bicocca, 20126, Milan, Italy; 6 Dept. of Molecular Cell Physiology, Faculty of Earth and Life Sciences, VU University, Amsterdam, The Netherlands; 7 Manchester Centre for Integrative Systems Biology, School of Chemical Engineering and Analytical Science, University of Manchester, Manchester, United Kingdom; 8 Swammerdam Institute for Life Sciences, Faculty of Science, University of Amsterdam, Amsterdam, The Netherlands; Christian Albrechts Universitat zu Kiel, GERMANY

## Abstract

Metabolic reprogramming is a general feature of cancer cells. Regrettably, the comprehensive quantification of metabolites in biological specimens does not promptly translate into knowledge on the utilization of metabolic pathways. By estimating fluxes across metabolic pathways, computational models hold the promise to bridge this gap between data and biological functionality. These models currently portray the average behavior of cell populations however, masking the inherent heterogeneity that is part and parcel of tumorigenesis as much as drug resistance. To remove this limitation, we propose single-cell Flux Balance Analysis (scFBA) as a computational framework to translate single-cell transcriptomes into single-cell fluxomes. We show that the integration of single-cell RNA-seq profiles of cells derived from lung adenocarcinoma and breast cancer patients into a multi-scale stoichiometric model of a cancer cell population: significantly 1) reduces the space of feasible single-cell fluxomes; 2) allows to identify clusters of cells with different growth rates within the population; 3) points out the possible metabolic interactions among cells via exchange of metabolites. The scFBA suite of MATLAB functions is available at https://github.com/BIMIB-DISCo/scFBA, as well as the case study datasets.

## Author summary

Cytotoxicity of chemotherapeutic agents and resistance to targeted treatments are the main reasons why cancer is still one of the top causes of death. As tumor cells are intrinsically resistant to therapies that target signaling pathways, targeting the metabolic hallmarks of cancer holds promise for more incisive treatments. Regrettably, the heterogeneity of cancer metabolism hinders the identification of effective treatments. To fully uncover the metabolic heterogeneity within tumors, characterization of metabolic programs (metabolic flux distributions) at the single-cell level is required. To fill the gap between current technologies for genomics and future technologies for fluxomics, both at the single-cell and the genome-wide scale, we propose to integrate cancer data from: 1) single-cell transcriptomics and 2) bulk metabolomics, into a multi-scale stoichiometric model, to deliver for the first time metabolic fluxomes at the single-cell level. To this end, we introduce a new paradigm for flux balance analysis and data integration in cancer metabolism to: 1) characterize metabolic heterogeneity, not only at the inter-, but also at the intra-tumor level 2) identify the metabolic interactions between cancer populations, whose role in resistance to metabolic treatments has been recently recognized 3) predict the collective response to drug targeting of metabolism.

## Introduction

Cancer is a heterogeneous, multi-factorial and essentially genetic disease, in which various types of mutations alter the functioning and interactions of genes, causing cancer cells to proliferate in an uncontrolled manner. Despite the plethora of cancer-related mutations, a reduced number of recognizable phenotypic hallmarks [1, 2] have been identified.

Metabolic rewiring, in particular, is a general feature of cancer cells, which reprogram their metabolism to feed their unrestrained proliferation, as it requires high amounts of energy and building-blocks [3]. The design principles underlying the causative role of metabolism in promoting growth as a function of the nutritional constraints are starting to be investigated [4, 5] and the idea of targeting the distinctive features of cancer metabolism has received considerable attention [6].

Unfortunately, a single metabolic program cannot be used to globally define an altered tumour metabolism [7], as cancer cells, even within the same tumour, may cope with the above metabolic requirements by engaging different metabolic pathways [8]. Such variability produces different dependencies on exogenous nutrients, and reflects into heterogeneous responses to metabolic inhibitors [9].

Furthermore, in solid tumours, cancer cells are embedded within the tumour microenvironment (TME), a complex network of fibroblasts, myofibroblasts, myoepithelial cells, vascular endothelial cells, cells of the immune system and extracellular matrix. TME also includes chemical gradients of oxygen and nutrients: the complex interaction of all these elements plays a major role in tumour metabolic heterogeneity [10]. The metabolic interplay that occurs among cancer cells and TME—supported by experimental evidence on how malignant cells may extract high-energy metabolites (e.g., lactate and fatty acids) from adjacent cells [11, 12]—contributes to treatment resistance [13]. Therefore, effective therapeutic strategy should incorporate knowledge of intra-tumour metabolic heterogeneity and cooperation phenomena within cancer cell populations.

Knowledge about the utilization of metabolic pathways requires quantification of metabolic fluxes (i.e., the rate at which a substance is transformed into another through a given reaction or pathway). As quantification of the full complement of cell metabolites (metabolomics) alone does not provide information on internal fluxes [14], they can be measured only indirectly, mainly via several isotope-labeled metabolomics experiments coupled with metabolic flux analysis. A more functional option is Flux Balance Analysis (FBA), which uses linear algebra algorithms to solve a mass balance problem, given constraints on the flux of some relevant reactions, with particular regard to extracellular fluxes (rate of intake and secretion of metabolites) [15]. An advantage of FBA is that, as opposed to intracellular fluxes, extracellular fluxes can also be approximated from measurements of the concentration of metabolites in the spent cell culture medium at different time points. FBA has proved able to correctly predict the growth yield of microorganisms when extracellular fluxes are constrained [16–18]. Several approaches have been proposed to set further constraints on internal fluxes, by exploiting other -omics data, such as transcriptomics or proteomics data. Protein abundances should be used cautiously as proxy for flux rates, as the availability of substrates ultimately determine the reaction rate. Transcript levels should be used even more prudently than protein abundances, because many factors beyond transcript concentration contribute to determine the expression level of a protein, such as translation and degradation rates, spatial locations and post-transcriptional regulation. However, the differences between the mRNA level observed in different conditions (at the steady state) explain most of the variation in concentration of the associated proteins [19, 20]. Therefore, coupling the information of transcripts with that on extracellular fluxes, within the FBA steady-state modeling framework, provides a reasonable solution to the problem of predicting intracellular fluxes.

Nevertheless, extracellular fluxes can be hardly measured at the single-cell level. They cannot be approximated from the spent cell culture medium, because it portrays the extracellular fluxes of the bulk. They might be measured with isotope-labeling and metabolic flux analysis techniques, but this would require single-cell metabolomics.

Unfortunately, single-cell metabolomics is still at its embryonic stage, mainly because of limitations in working with minute amounts of material [21–23]. This is a major problem, because flux distributions are currently estimated from metabolic measurements retrieved from bulk samples, which often contain intermixed and heterogeneous cell subpopulations, thus overlooking possible cooperation and compensation phenomena. In the simplest example, let us imagine that two populations exist: one that secrete lactate and another that consumes all the lactate produced by the former. The predicted flux distribution will represent the sum of the two populations, hence the flux through lactate production/consumption will be regarded as inexistent, portraying a behavior far from the real one. This limitation might partially be overcome by analyzing a subpopulation of cells supposedly having a more homogeneous metabolism, for example, by using fluorescence-activated cell sorting techniques to isolate it according to specific physical properties. However, when dealing with populations of cells derived from tumours, it is difficult to assess the relative composition of intermingled cancer cells and of stromal elements within the tumor architecture, and fluorigenic markers may not robustly correlate with metabolic phenotype. Hence, stratification, in terms of metabolic function and of consequent susceptibility to metabolic drugs, of heterogeneous populations taken, for example, from biopsies, xenografts or organoids, requires characterization of cellular metabolism at the single-cell level.

At this purpose, we here introduce the first computational framework scFBA (single-cell Flux Balance Analysis) to predict single-cell fluxomes and possible metabolic interactions among them, starting from (bulk) extracellular fluxes and single-cell transcriptomes. As compared to single-cell metabolomics, the field of single-cell transcriptomics has indeed progressed to a deeper resolution [5, 24].

An established classification [25–29] of current methods to integrate (bulk) transcriptomic data into constraint-based models is based on the purpose of the method, and distinguishes approaches that aim at extracting the active metabolic network (typically from a genome-scale one, e.g., [30–33]) from methods that aim at estimating the extent of the flux of each reaction in a network (e.g., [34–39]). However, these methods cannot be directly extended to single-cell modeling, because of the lack of information of extracellular fluxes at the single-cell level discussed above. Even if it was possible to measure single-cell extracellular fluxes, there is no protocol to measure transcriptome and fluxes of the very same single-cell (the cell is destroyed after either analyzing the trascriptome or metabolome). For this reason, we propose to use a multi-scale model, in order to solve a unique mass balance problem to identify the possible combination of single-cell steady states that concurrently satisfies constraints on single-cell transcriptomes and extracellular bulk fluxes. In order to determine the flux distribution of each network, each cell is not considered in isolation: it is allowed to interact with other cells in the population, via release/uptake of metabolites into/from the TME.

Datasets obtained from cancer biopsies or patient-derived xenografts are the ideal candidate to capture the heterogeneous composition of tumors. As currently no information on scRNA-seq and extracellular fluxes on the very same sample is publicly available, as a proof of principle, we here applied scFBA to lung adenocarcinoma (LUAD) patient derive xenograft scRNA-seq, collected by [40], while testing different sets of constraints for the extracellular fluxes. To prove the robustness and applicability of our method, we also applied scFBA on further independent breast cancer datasets collected by [41].

## Methods

### Approach

Although some attempts to study the cooperation between different metabolic populations have been put forward [42–44], mostly focused on microbial communities, these methods require indeed *a priori* knowledge about the specific metabolic requirements and objectives of the intermixed populations. Unfortunately, even though metabolic growth may approximate the metabolic function of some cell populations, we cannot assume that each cell within an *in vivo* cancer population proliferates at the same rate, nor that it proliferates at all. A major example is given by the different proliferation rates of stem and differentiated cells [45]. For this reason, differently from other approaches [44], we do not impose that the population dynamics is at steady-state (and hence that cells all grow at the same rate), although we do continue to assume that the metabolism of each cell is. Conversely, scFBA aims at portraying a snapshot of the single-cell (steady-state) metabolic phenotypes within an (evolving) cell population at a given moment, and at identifying metabolic subpopulations, without *a priori* knowledge, by relying on unsupervised integration of scRNA-seq data.

We have previously shown how Flux Balance Analysis of a population of metabolic networks (popFBA) [46] can in line of principle capture the interactions between heterogeneous individual metabolic flux distributions that are consistent with an expected average metabolic behavior at the population level [46]. However, the average flux distribution of a heterogeneous population can result from a large number of combinations of individual ones, hence the solution to the problem of identifying the actual population composition is undetermined. To reduce this number as much as possible, we here propose to exploit the information on single-cell transcriptomes, derived from single-cell RNA sequencing (scRNA-seq), to add constraints on the single-cell fluxes.

An identical copy of the stoichiometry of the metabolic network of the pathways involved in cancer metabolism is first considered for each single-cell in the bulk. To set constraints on the fluxes of the individual networks, represented by the single-cell compartments of the multi-scale model, we took inspiration from bulk data integration approaches that aim to improve metabolic flux predictions, without creating context-specific models from generic ones [34–39]. At the implementation level, we use continuous data, rather than discrete levels, to overcome the problem of selecting arbitrary cutoff thresholds. At this purpose, some methods (e.g. [30, 32]) use expression data to identify a flux distribution that maximizes the flux through highly expressed reactions, while minimizing the flux through poorly expressed reactions. To limit the problem of returning a flux distribution (or a content-specific model) that does not allow to achieve sustained metabolic growth, we use instead the “pipe capacity” philosophy embraced by other methods, such as the E-Flux method [36, 37], of setting the flux boundaries as a function of the expression state. These methods tend to use relative rather than absolute expression values. For instance, the original formulation of E-flux [36] sets relative boundaries in relation to the most expressed reactions. In order to avoid comparing enzymes with different gene-protein translation rates, which may also largely differ in their kinetic parameters (e.g. binding affinity) and in the number of associated isoforms/subunits, we prefer to normalize boundaries in relation to the condition/cell/tissue in which a given reaction is mostly expressed, as done in a more recent version of the E-flux method [37] and in other continuous methods [34, 35, 47]. Specifically, we distribute the total (bulk) possible flux of each reaction proportionally to the activity score of that reaction in each cell. To compute a score for reactions that involve many genes, similarly to other approaches [36–38, 48], we assume that enzyme isoforms contribute additively to the overall activity of a given reaction, whereas enzyme subunits limit its activity, by requiring all the components to be present for the reaction to occur. Alternative but similar approaches, such as [30], consider the maximum value (instead of the sum) of isoform values.

**Fig 1:**
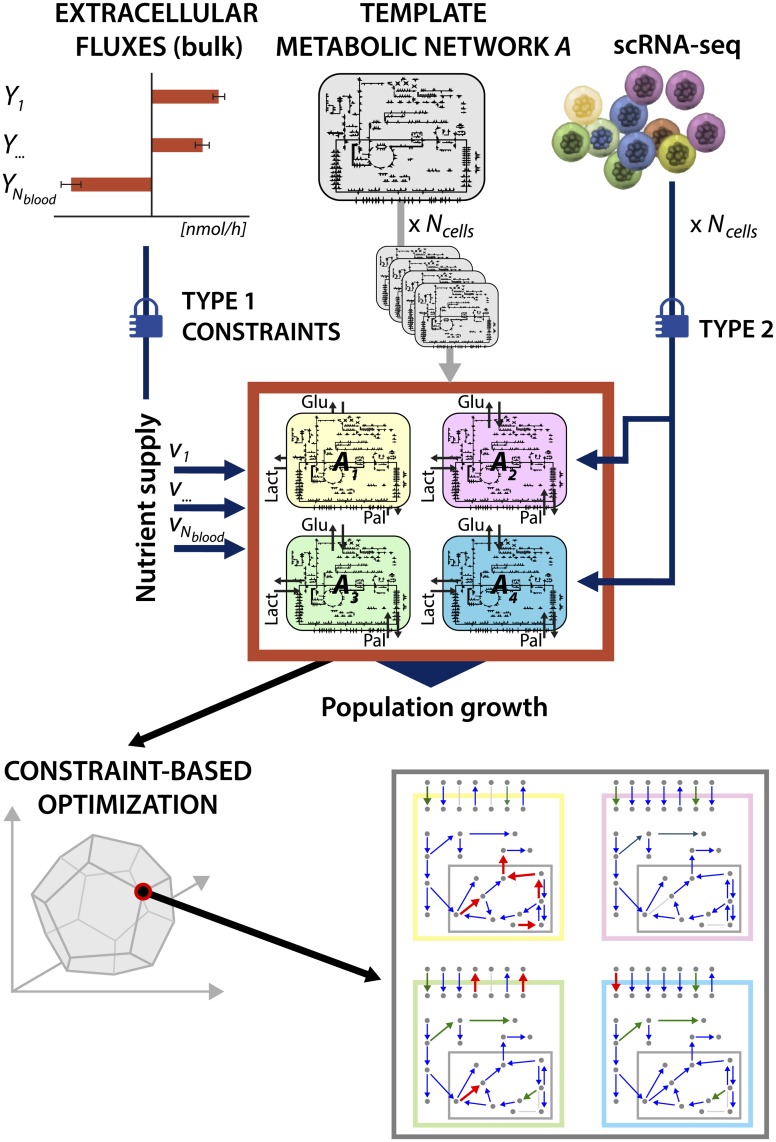
Graphical representation of scFBA. Extracellular fluxes and sc-transcriptomes are translated respectively into type 1 and 2 heterogeneous constraints (see Materials and methods) imposed on a initially homogenous population of *N*_cells_ replicates of metabolic network *A*. The output is a heterogeneous set of flux patterns that may predict sc-fluxes.

A scheme of the scFBA approach is depicted in Fig 1.

### popFBA

Here we briefly recall the popFBA approach. For a more comprehensive description, the reader is referred to [46].

Starting from a template metabolic network map *A*, corresponding to a generic single-cell and defined as $A=(X^{A},R^{A},E^{A})$—where $X^{A}=\left\{X_{1},\ldots ,X_{M}\right\}$ is the set of metabolites in network *A*, $R^{A}=\{R_{1},\ldots ,R_{N}\}$ the set of biochemical reactions taking place among them and $E^{A}=\{E_{1},\ldots ,E_{N_{\text{ext}}}\}$ is a set of *N*_ext_ unbalanced reactions (exchange reactions), enabling a predefined set of metabolites (including the pseudo-metabolite representing biomass) $Y=\left\{Y_{1},\ldots ,Y_{N_{\text{ext}}}\right\}\subset X^{A}$ to be inserted in or removed from the system—the popFBA procedure first builds a population model composed of *N*_cells_ replicates *A*^*c*^ of network *A*, each one corresponding to a single-cell *c*, *c* = 1, …, *N*_cells_, and which can cooperate by exchanging nutrients in the tumour microenvironment.

For each single-cell *c*, $A^{c}=\left(X^{c},R^{c},C^{c}\right)$ is its metabolic network, where:

- $X^{c}\equiv X^{A}$ is the set of its metabolites;
- $R^{c}\equiv R^{A}$ is the set of its internal reactions;
- $C^{c}=\left\{C_{j}^{c}\right\}$, with *j* = 1, …, *N*_ext_, is a set of cooperation reactions, defined as reactions that allow to exchange metabolites among single-cells via a shared environment that represents the TME compartment. Cooperation reactions are built by transforming each exchange reaction $E_{j}\in E^{A}$ into a cooperation reaction $C_{j}^{c}$ with the form:
  $$\begin{array}{c} C_{j}^{c}:Y_{j}^{c}\;↔\;Y_{j}^{\prime } \end{array}$$ (1)

Accordingly, a new set of metabolites pertaining to the TME compartment $Y^{\prime }=\left\{Y_{i}^{\prime }\right\}$ with *i* = 1, …, *N*_ext_ must also be defined.

Because original exchange reactions have been replaced by cooperation reactions, a new set of *N*_blood_ exchange reactions $B=\{B_{1},\ldots ,B_{N_{\text{blood}}}\}$ is defined, which allows a subset of metabolites $K=\left\{K_{1},\ldots ,K_{N_{\text{blood}}}\right\}\subset Y^{\prime }$ to be exchanged with the external environment, e.g., the blood supply:

$$\begin{array}{c} B_{j}:K_{j}\;↔\;\emptyset \end{array}$$ (2)

The population model *P* is then defined by: (*i*) the union set of the metabolites $X^{P}=\cup_{c}X^{c}\cup Y^{\prime }$; (*ii*) the internal reactions $R^{P}=\cup_{c}R^{c}$; (*iii*) the cooperation reactions $C^{P}=\cup_{c}C^{c}$; (*iv*) the population exchange reactions B.

A stochiometric matrix *S*^*P*^ is then built for all reactions in $R^{P}$, $C^{P}$ and B and for all metabolites in $X^{P}$ and $Y^{\prime }$. The final size of matrix *S*^*P*^ is (*N*_cells_ ⋅ *M* + *N*_blood_) × (*N*_cells_ ⋅ (*N* + *N*_ext_) + *N*_blood_). Once the population model is obtained, the total biomass of the *N*_cells_ single-cells is maximised by means of linear programming, as in standard FBA [15].

The solution of popFBA represents the flux distribution $\vec{v}=\left(v_{1},\ldots ,v_{N_{\text{cells}}\cdot \left(N+N_{\text{ext}}\right)+N_{\text{blood}}}\right)=\left(r_{1}^{1},\ldots ,r_{N}^{1},\ldots ,r_{1}^{N_{\text{cells}}},\ldots ,r_{N}^{N_{\text{cells}}},c_{1}^{1},\ldots ,c_{N_{\text{ext}}}^{1},\ldots ,c_{1}^{N_{\text{cells}}},\ldots ,c_{N_{\text{ext}}}^{N_{\text{cells}}},b_{1},\ldots ,b_{N_{\text{blood}}}\right)$ that maximises the biomass exchange flux *b*_*biomass*_, with *v*_*i*_ representing any flux *i* of the population model, and for each single-cell *c*, $r_{i}^{c}$ representing the *i*-th internal flux, $c_{i}^{c}$ representing the *i*-th cooperation flux and *b*_*i*_ an exchange flux with blood. The optimization problem is postulated as follows:

$$\begin{array}{c} \begin{array}{c} \text{maximise}\;b_{biomass}\\ \text{subject}\;\text{to}\;S\vec{v}=\vec{0},{\vec{v}}_{L}\leq \vec{v}\leq {\vec{v}}_{U} \end{array} \end{array}$$ (3)

${\vec{v}}_{L}$ and ${\vec{v}}_{U}$ are vectors specifying the lower and upper bound respectively for each flux *v*_*i*_ of $\vec{v}$. A negative lower bound indicates that flux is allowed in the backward reaction. To solve the above problem we exploited the Gurobi solver within the COBRA Toolbox [49].

### Input and data pre-processing

scFBA takes as input a template metabolic network map A, as in popFBA, plus a scRNA-seq dataset in the form of a *N*_genes_ × *N*_cells_ matrix *T*, where *N*_genes_ is the number of genes and *N*_cells_ is the number of single-cells under study. Each element *T*_*g*,*c*_, *g* = 1, …, *N*_genes_, *c* = 1, …, *N*_cells_ corresponds to the normalized read count of gene *g* in cell *c* such as, for instance, the TPM (Transcripts Per Kilobase Million).

The risk of the presence of false negatives in RNA-seq, and in particular scRNA-seq, is an established problem. Although a totally safe solution does not exist, scFBA allows to employ the information on bulk expression profile, when available, to manage the risk, by envisioning the following scenarios.

- If a gene has a zero read count in the bulk, as well as in each single-cell, we cannot totally exclude the possibility of a false-negative in the bulk, but we are confident in excluding a false-negative due to low concentrations of scRNA-seq, thus we can assume that such gene is off in all cells. We directly delete this set of genes *G*_*off*_ from the template metabolic network *A*, by solving the Gene-Protein-Reaction (GPR) association rules with a true-false logic (Cobra Toolbox [49] function: *geneDeletionAnalysis*), which results in removing reactions for which their expression is essential (*AND* operator). We refer to the obtained subnetwork of *A* as A*.
- If a gene has non-zero read count in the bulk, but a zero read count in each single-cell, there is a sharp inconsistency between bulk and scRNA-seq that indicates that we cannot trust scRNA-seq for this gene. In this situation, we prefer to lose information on single-cell heterogeneity and rely on the bulk value: we replace the read count for that gene in each cell with the bulk read count.
- If a gene has non-zero read count in the bulk, and zero read count in some of the single-cells, we cannot be sure that the gene is actually not expressed in those cell, but we can exclude that there is a problem with the detection of that specific gene and we can hypothesize that is at least poorly expressed as compared to other cells. As a compromise between a more conservative strategy and the need to preserve information on cell heterogeneity, we retain the single-cell read count for these genes, but we do not prevent completely flux through the associated reactions, when setting boundaries of the reaction as a function of their expression. As we will illustrate in the following, we set the flux bound to a small value *ϵ*.

### Reaction activity scores

We define a Reaction Activity Score (RAS), for each single-cell *c* = 1, …, *N*_cells_, and each reaction j∈R, based on Gene-Protein-Reaction association rules (GPRs). GPRs are logical formulas that describe how gene products concur to catalyze a given reaction. Such formulas include AND and OR logical operators. AND rules are employed when distinct genes encode different subunits of the same enzyme, i.e., all the subunits are necessary for the reaction to occur. OR rules describe the scenario in which distinct genes encode isoforms of the same enzyme, i.e., either isoform is sufficient to catalyze the reaction.

In order to compute the RAS we distinguish:

- Reactions with AND operator (i.e., enzyme subunits).
  $$\begin{array}{c} RAS_{j}^{c}=\min_{g}\left(T_{g,c}:g\in S_{j}\right) \end{array}$$ (4)
  where *S*_*j*_ is the set of genes that encode the subunits of the enzyme catalyzing reaction *j*.
- Reactions with OR operator (i.e., enzyme isoforms).
  $$\begin{array}{c} RAS_{j}^{c}=\sum_{g\in I_{j}}T_{g,c} \end{array}$$ (5)
  where *I*_*j*_ is the set of genes that encode isoforms of the enzyme that catalyzes reaction *j*.

In case of composite reactions, we respect the standard precedence of the two operators.

### scFBA

The first step of the scFBA approach is the creation of a multi-scale population model, composed of *N*_cells_, according to the popFBA described above, but starting from the template metabolic network A*. As described in the subsection related to input and data pre-processing, A* is a subnetwork of the generic model *A* which integrates the transcriptional information that holds for all cells in the bulk.

Once the population model is obtained, the scFBA approach imposes two kinds of constraints:

type 1 constraints on the extracellular fluxes of the overall population model *P*, i.e., the upper and lower bound of the *N*_blood_ exchange reactions in set B, ideally according to metabolic measurements;
type 2 constraints on internal fluxes of each single-cell *c*, i.e. for the reactions in $R^{c}$, with *c* = 1, …, *N*_cells_, and for its set of cooperation reactions *C*^*c*^, according to their RAS, whenever the computation of a RAS is possible, i.e., when a GPR exists for the reaction, along with the transcript values of at least one of the involved genes.

In order to project the information of the activity score of a given reaction *j*, in a given cell *c*, $R_{j}^{c}$, onto its flux “pipe capacity”:

- we first estimate the possible flux that reaction $R_{j}^{c}$ might carry, when only constraints on extracellular fluxes (type 1) are set, whereas the internal fluxes (type 2) are still unbounded and the system is not required to make biomass, i.e., we compute the maximal flux in both the forward (*F*_*f*_) and backward direction (*F*_*b*_) of each reaction. To do so, we perform a Flux Variability Analysis [50], with no optimality required (Cobra Toolbox [49] function: *fluxVariability*). We define $F_{j}^{c}=\max(|F_{f}\left|,\right|F_{b}\left|\right)$.
- we then compute the relative reaction activity score of $R_{j}^{c}$ in each *c* = 1, …, *N*_cells_, with respect to the total activity of reaction *j*, as follows:
  $$\begin{array}{c} {\bar{RAS}}_{j}^{c}=\frac{RAS_{j}^{c}}{\sum_{c=1}^{N_{\text{cells}}}RAS_{j}^{c}}, \end{array}$$ (6)
- finally, we assign an upper bound ($U_{j}^{c}$) to reaction $R_{j}^{c}$, as portion of $F_{j}^{c}$ which is proportional to the activity score (${\bar{RAS}}_{j}^{c}$) of reaction $R_{j}^{c}$. Namely, we remap the values $RAS_{j}^{c}$, *j* = 1, …, *N*;*c* = 1, …, *N*_cells_ in the interval $\left[\in ,F_{j}^{c}\right]$, as follows:
  $$\begin{array}{c} U_{j}^{c}=\epsilon +\left(F_{j}^{c}-\epsilon \right)\cdot {\bar{RAS}}_{j}^{c} \end{array}$$ (7)
  We set the upper bound of reactions having ${\bar{RAS}}_{j}^{c}=0$ to a small value *ϵ* rather than to 0 to mitigate the impact of false-negatives. Note that $\sum_{c=1}^{N_{\text{cells}}}U_{j}^{c}\approx F_{j}^{c}$. We remind that the set of genes *G*_*off*_ is instead fully deleted from the model. As baseline value, in this study we set ∈ = 10^−3^, but we assess how its variation may affect the results by scanning the values: {0, 10^−6^, 10^−5^, 10^−4^, 10^−3^, 10^−2^, 10^−1^, 1}.
- if reaction $R_{j}^{c}$ is considered irreversible, we assign a zero lower bound ($L_{j}^{c}=0$) to reaction $R_{j}^{c}$, otherwise we assign a lower bound $L_{j}^{c}=-U_{j}^{c}$. The reason why, when dealing with reversible reactions, we avoid setting different values for backward and forward reaction, by assigning to $F_{j}^{c}$ the maximum value between *F*_*f*_ and *F*_*b*_, is that the RAS reflects the gene expression of its competent enzyme, which may equally work in either direction.
  Once the *P* model is constrained (with both type 1 and type 2 constraints), Linear Programming, as well as other standard constraint-based methods were applied.

### Datasets

In this work, we mainly use the following 3 LUAD datasets obtained from the NCBI Gene Expression Omnibus (GEO) data repository under accession number GSE69405.

**LCPT45** Composed of 34 cells acquired from a xenograft, obtained by sub-renal implantation in mice of a surgical resection of a 37-mm irregular primary lung lesion in the right middle lobea of a 60-year-old untreated male patient.
**H358** Composed of 50 cells from NCI-H358 bronchioalveolar carcinoma cell line.
**LCMBT15** Composed of 49 cells acquired from a xenograft, obtained by sub-renal implantation in mice of a surgical resection of a metachronous brain metastasis acquired from a 57-year-old female after standard chemotherapy and erlotinib treatments.

We repeated all the analyses on the following independent breast cancer datasets (GEO access number: GSE75688), including scRNA-seq data of single-cell suspensions of cancer tissues obtained on the day of the surgery of untreated breast cancer patients [41]:

**BC04** Composed of 59 human epidermal growth factor receptor 2 positive (HER2+) cells.
**BC03LN** Composed of 55 lymph node metastases of human estrogen receptor positive (ER+) and human epidermal growth factor receptor 2 positive (HER2+) cells.

Each of the 5 datasets includes the gene expression level of more than 20.000 genes in the form of Transcript Per Kilobase Milion (TPM). We filtered out a few cells with less than 5000 genes detected. For each dataset, we retained only the metabolic genes included in HMRcore model (418 genes). The dataset transcripts are identified by Ensembl ID, which we automatically converted into HUGO Gene Nomenclature Committee (HGNC) ID. The datasets also contain the expression profile of the bulk samples, which we used to pre-process data as described above.

### Metabolic network model

From the computational perspective, the scFBA approach is suitable for simulation of genome-wide metabolic networks, such as [51, 52]. However, in view of previous analyses [4], in order to have more control on the analyses and make the interpretation of results more straightforward, at this stage, we preferred to focus on a more handful and carefully reconstructed core metabolic network. We used, as template network *A*, the metabolic core model HMRcore introduced in [53] and used in [46, 48]. As exchange of fatty acids between cells in tumours has been recently reported [12, 54], we included the possibility to exchange palmitate via the TME and, accordingly, mitochondrial palmitate degradation and gluconeogenesis. Given the importance of reactive oxygen species (ROS) metabolism observed in [4], we also inserted ROS production and removal pathways. As the original version of the model does not include information on GPRs, such rules have been extracted from Recon 2.2 [51] and included in the HMRcore model. We decided to disregard the GPRs associated to the complexes I to IV of the electron transport chain in scFBA computations, because it unrealistically requires up to 81 genes (AND rule). However the flux through complexes I to IV should be modulated by the constraints on complex V (ATP synthase).

The final version of the HMRcore model includes 315 reactions (of which 263 are associated with a GPR) and 418 metabolic genes. The SBML of the model is provided in https://github.com/BIMIB-DISCo/scFBA.

#### Experimental setting

The choice of the nutrients exchanged with biofluids should ideally be dictated by metabolic measurements on exo-metabolome. As we do not have this information, in the baseline experimental setting, we considered as main exogenous nutrients (which the overall population can uptake from 0 up to *Npop* ⋅ 100 *nmol*/*h*) those that are the main nutrients of cancer cells according to literature, as motivated in [4]: glucose, glutamine, oxygen: glucose, glutamine, oxygen and arginine. Along similar lines, we considered as nutrients that can be secreted by cancer cells in the tumor microenvironment those that are mainly reported in literature, and which may play a role in metabolic cooperation: glutamate [55], *NH*_3_ [56–58], lactate [59–61], and palmitate [12, 54]. In order to be able to discern the advantage of cooperation from that of the mere secretion of metabolites, we considered both a cooperation reaction and a secretion reaction for these nutrients.

## Results

### Integration of RNA-seq data efficiently reduces the space of optimal solutions

We first applied scFBA to the 5 datasets described in the Methods section, assuming maximization of total (population) biomass synthesis rate as objective function. All five population models displayed a non negligible maximal growth rate, something that cannot be taken for granted when integrating transcriptomics into FBA models [25, 27, 29]. In S1 Fig and accompanying S1 Text, we also show that, if *ϵ* takes value 0, the scFBA problem is still feasible, but we obtain very small values for the fluxes.

**Fig 2:**
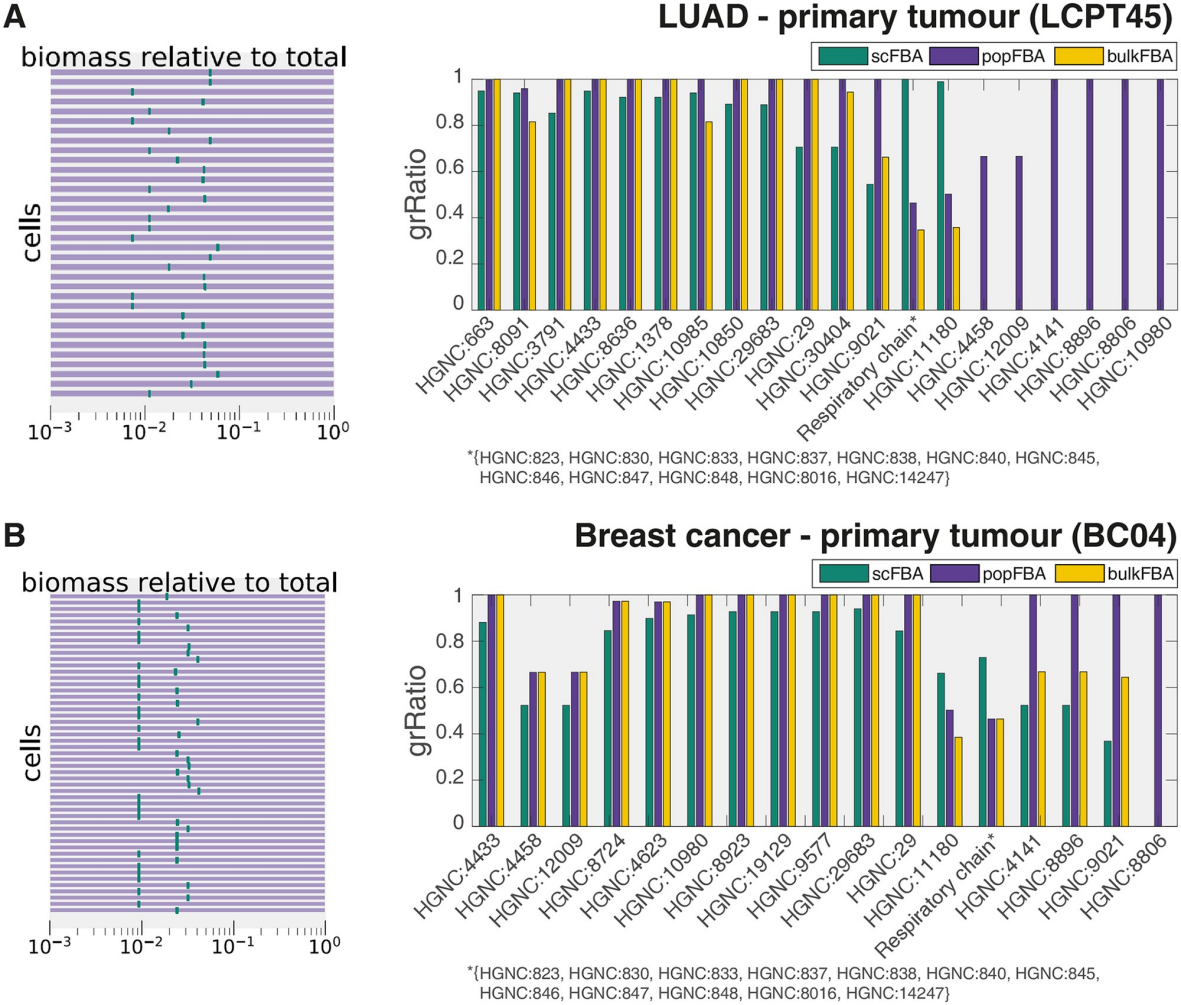
scFBA vs. popFBA. A) Dataset LCPT45. Variability of the fraction of the biomass synthesis flux (logarithmic scale) for each cell over the population growth rate (left panel) before (purple) and after data integration (green). Effect of gene deletion (bars in right panel) on the population growth rate before (popFBA), after data integration (scFBA), and for the template metabolic network A* (bulkFBA). When *grRatio* = 0 (essential gene), the corresponding bar is not displayed. B) Same information as in A for BC04 dataset.

In order to highlight that the scFBA approach efficiently reduces the space of optimal solutions, we compared the variability of the biomass production flux of each of the *N*_cells_ single-cells simulated within the population model, for each of the 5 datasets under study. We report in Fig 2 the results for the two datasets relative to the primary tumors, and in S2 Fig for the other datasets.

When no information on cells’ transcriptome is employed (as in standard popFBA settings [46]), the type 2 constraints of the metabolic network are identical for all cells. This implies that each cell is capable of contributing alone to 100% (10^0^) of the objective function value (i.e., the biomass of the total population). As depicted in Fig 2 (left plots) and S2 Fig (left plots), the biomass flux value of each cell, within the set of optimal solutions, spans indeed from 0 to 100% of the total biomass (purple rectangles). On the contrary, after scRNA-seq data integration, as performed via scFBA, the biomass flux of each cell can only take a specific (optimal) value, corresponding to a certain fraction of the total biomass (green rectangles, which results in a single line, because the maximum and minimum optimal flux values coincide.)

To show how this volume reduction in the space of alternative optima may actually affect predictions, we performed a single gene deletion analysis with and without scRNA-seq data integration (scFBA and popFBA, respectively). When a single gene is deleted, the reactions for which the expression of such gene is essential (i.e., reactions exclusively associated to the gene, or reactions associated to that gene and other genes with an AND operator) are removed from the network (i.e., from the set $R^{c},\forall c$). After removal, the population model is newly optimized for total biomass production, and the growth ratio (*grRatio*) of the new biomass over the previous one is computed.

Fig 2 and S2 Fig (right bar plots) report the *grRatio* observed for those genes deletions that displayed a different effect before and after data integration. Notice that when the *grRatio* equals 0, the corresponding bar is not displayed at all. To verify that the differences between scFBA and popFBA are not a mere consequence of the removal of reactions (in scFBA) that are inactive in all cells of the bulk from the template metabolic network *A*, we include in the plots the prediction of the isolated template metabolic network A*. We refer to this third simulation setting as bulkFBA. However, bulkFBA includes information on on-off reactions only. It is not possible to modulate the flux capacity of reactions as a function of gene expression, because it not possible to compute relative expression values.

Remarkably, some genes that are redundant (*grRatio* = 1) in popFBA settings (i.e., with no scRNA-seq data integration) may even become essential in scFBA settings (i.e., with scRNA-seq data integration) (*grRatio* = 0). This is the case, in lung adenocarcinoma, of the following genes: HGNC:10980, which encodes enzymes responsible for glutathione/phosphate, fumarate/phosphate or *α*-ketoglutarate/malate antiports; HGNC:8806, which encodes for a subunit of pyruvate dehydrogenase; HGNC:8896, which encodes for an isoform of phosphoglycerate kinase and HGNC:4141, which encodes for an isoform of glyceraldehyde 3-phosphate dehydrogenase. In breast cancer, only gene HGNC:8806 falls into this category. Conversely, some genes that display a significant effect (*grRatio* ≈ 0.5) in popFBA become instead redundant in scFBA. This is the case, in lung adenocarcinoma, of the genes that encode for ATP synthase (HGNC:823, 830, 833, 837, 838, 840, 845-848, 14247, 8016), indicating that the integration of scRNA-seq data forces a (suboptimal) flux distribution for cancer cells which, consistently with the well-known Warburg effect, does not rely largely on ATP synthase for ATP production, thus resulting in a milder effect when the reaction is depleted. Worth of note, although these genes are not completely redundant (*grRatio* < 1) in breast cancer, the deletion of the respiratory chain has indeed a mild effect in both tumors, as well as in the other cancer datasets reported in S2 Fig.

It is apparent, from Fig 2 and S2 Fig (right bar plots), that bulkFBA provides intermediate results between scFBA and popFBA. Some genes that are redundant in PopFBA, are lethal in both scFBA and bulkFBA. This is the case for example in LCPT45 of gene HGNC:10980 (Mitochondrial dicarboxylate carrier). This result is expected, given that its isoform has been deleted according to bulk data in both simulations. Conversely, some gene deletions that have a significant effect according to bulk data have no effect when also single-cell data are considered, in particular the genes encoding for components of the respiratory chain. Also, the effect of the deletion of pyruvate kinase (HGNC:9021) is smaller in scFBA than in bulkFBA. On the other hand, some deletions may show some effect only when scRNA-seq are considered. This is particularly true for genes that are involved in cooperation mechanisms among cells, as for instance gene HGNC:29, in both datasets, whose product promotes the secretion of palmitate, which can be taken up by other cells.

### scFBA extracts useful features from transcript signals

As previously mentioned, single-cell fluxes are expected to be less noisy than transcript signals, which are typically analyzed by means of multi-variate statistical analysis [5] and, therefore, the former might be used to better identify cell clusters that might represent distinct metabolic subpopulations. To confirm this hypothesis, we performed a cluster analysis on the expression values (scRNA-seq) of the metabolic genes and compared the results with those of a cluster analysis performed on the fluxes predicted by scFBA. To this end, we performed both hierarchical and k-means cluster analysis. In order to avoid reactions with typical high flux-value, or genes with high expression, to induce a bias on clustering results, we first remapped the flux (transcript) values of each reaction (gene) *j* in the interval [0, 1]: value 0 is assigned to the cell showing the lowest value for a given flux (transcript), 1 to the one showing the highest value.

**Fig 3:**
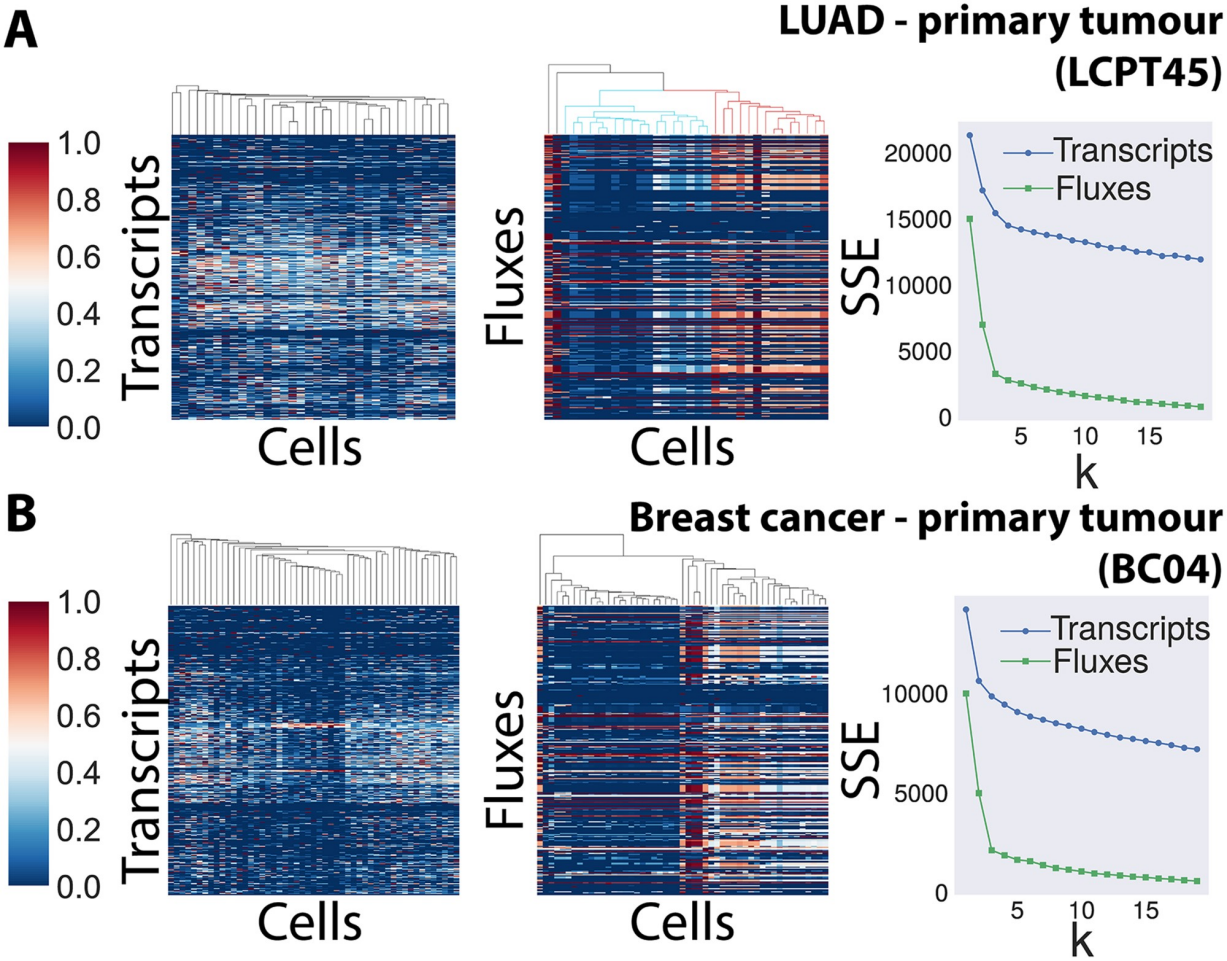
Clustering of transcripts vs. fluxes. A) LCPT45 dataset. Clustergram (distance metric: euclidean) of the transcripts of the metabolic genes included in metabolic network (left) and of the metabolic fluxes predicted by scFBA (middle). Right panel: elbow analysis comparing cluster errors for *k* ∈ {1, ⋯, 20} (k-means clustering) in both transcripts (blue) and fluxes (green). B) Same information as in A for the BC04 dataset.

Fig 3 and S3 Fig report the results of the hierarchical clustering analysis (distance metric: euclidean), for transcripts (left column) and fluxes (middle column), respectively for the two primary tumors and for the other 3 datasets under study. From the dendrograms and heat-maps, one can see that cells cluster in a few well-separated groups, when the extracted features (the fluxes) are considered, whereas they cluster more in “singletons”, when the original features (the transcripts) are used. For example, when observing the fluxes computed for the LUAD dataset LCPT45 (panel A in Fig 3), it is apparent that two major (and a minor) groups of cells can be identified, corresponding respectively to the blue and red-coloured leaves in the dendrogram.

We evaluated which are the most different fluxes among the two major groups, by using the Z score test of statistical significance (S1 Table). Remarkably, the two groups significantly differ in their growth rates (Z-scores: 3.2). 82 reactions significantly differ between the two groups with a 99% confidence level. This set mostly include pathways directly or directly linked with biomass synthesis, such as biosynthesis of fatty, amino and nucleic acids.

To quantitatively compare the clustering of transcripts and fluxes, we first performed a k-means clustering with different number of clusters *k*, by considering *n* = 100 bootstrap iterations (with random centroid assignments) and by selecting the clustering resulting in the maximum inter-cluster distance. We then assessed the clustering goodness, by means of traditional “elbow” and “silhouette” evaluation methods. We refer to S2 Text for details about these approaches.

The elbow method in the right column of Fig 3, indicates that a elbow is observed at *k* = 3 for the fluxes relative to the primary tumour datasets, hence the optimal number of clusters is 3, which corresponds indeed to the *k* identified by the hierarchical clustering analysis for these two datasets.

In S3 Fig (panel D), we evaluated the silhouette for the dataset LCPT45 transcripts (left) and fluxes (right) for *k* = 3, i.e., the value identified from the elbow analysis, which also corresponds to the highest average silhouette value, when varying *k* in {2, ⋯, 6}.

Consistently with the more ready recognition of major clusters in the flux diagrams noted above, the drop in the sum of squared errors (SSE) is much stronger and the average silhouette value is considerably higher in the flux case than in the transcripts case (where the average coefficient is close to 0) indicating that the calculation of fluxes leads to a better clustering as compared to the evaluation of transcripts. All in all, the results of the cluster analyses indicate that fluxes can be better clustered than their transcript counterparts. Remarkably, it can also be noticed that the LUAD primary tumour xenograft (see the sharper “elbow” and the clustergram in Fig 3A) fluxes better partition into clusters than the fluxes of the cell line (S3 Fig, H358) and of the secondary tumour xenograft (S3 Fig, LCMBT15). This result is in line with the data reported in [40], indicating that a binary separation of cells is more evident in LCPT45. We detected a similar difference between the clustering results of primary and secondary tumour of the independent breast cancer datasets (BC04 in Fig 3 and BC03LN in S3 Fig). Indeed, the former population is more heterogeneous in the binary sense than the latter [41].

### scFBA captures interactions between cells

The main rationale behind solving a unique mass balance problem for many cells together, given constraints on the extracellular fluxes of the bulk, rather than many separate mass balance problems, is that the nutrient consumption and secretion rates (extracellular fluxes) can be readily determined or approximated from measurements of the concentration of metabolites in the cell culture media at different time points for the bulk only. Another major side benefit of this approach is that it allows to identify the possible interactions among cells within a population, as pointed out in [46].

**Fig 4:**
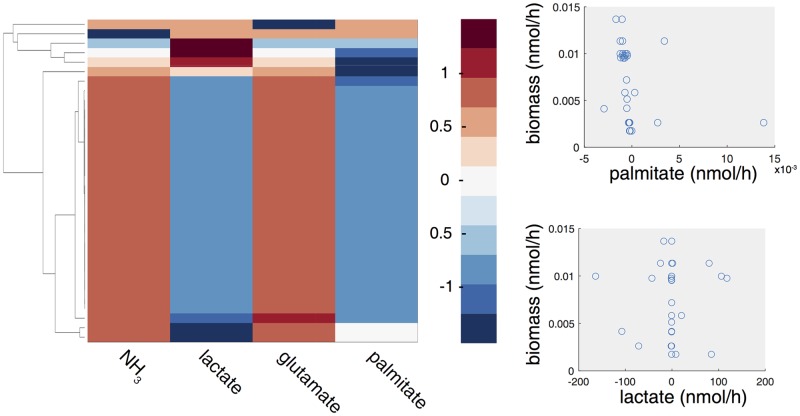
Metabolic cooperation in LCPT45 population. Left: Clustergram of the fluxes of cooperation reactions for NH_3_, lactate, glutamate and palmitate. Negative fluxes (blue shades) indicate an uptake, whereas positive fluxes (red shades) indicate a secretion of the corresponding metabolite. Right: Scatterplot of the biomass flux values of each cell in the population vs. palmitate (top) or vs. lactate cooperation flux (bottom).

We verified that, after data integration, some cells secret metabolites that are up-taken by other cells. The heat map in Fig 4 shows the (normalized) flux values of cooperation reactions for the LCPT45 dataset: a positive value means that the cell is secreting the metabolite in the tumour microenvironment, whereas a negative flux that the cell is uptaking it from the tumour microenvironment. It can be observed that a complex network of interactions is established among cells. In particular, a consistent group of cells consumes the lactate and palmitate that are secreted by other groups. The scatter plots in Fig 4 show the dispersion of the fluxes of uptake/secretion from/into the TME for lactate and palmitate and how they couple with different growth rates, portraying a relationship far more complex than that depicted with popFBA (no scRNA-seq integration [46] and no exchange of palmitate allowed). More in detail, the dominant subpopulation (larger cluster), which includes the majority of the cells, displays secretion of *NH*_3_ and glutammate and uptake of lactate and palmitate. Within the dominant subpopulation, only mild differences in flux rates can be detected, whereas within the other (smaller) subpopulations heterogeneous patterns of nutrient production/consumption are observed, which result from metabolic interactions.

It is also worth remarking that the predictions obtained with scFBA would not be possible with context-specific extraction methods from bulk data. In this respect, we compared the results of scFBA with two widely-used methods, GIMME [62] and iMAT [63], by focusing on the extracellular fluxes of the dominant single cell population identified with scFBA (see S2 Table for details). As expected, GIMME and iMAT cannot predict the consumption of certain metabolites, unless they are provided as exogenous nutrients. Indeed, both methods would not predict the consumption of lactate and palmitate. Even the prediction on produced metabolites may differ from that of the dominant population in scFBA: although both methods predict *NH*_3_ to be produced, consistently with scFBA predictions, glutamate would be produced according to iMAT only.

Metabolic interactions between cancer-associated fibroblasts and cancer cells, mediated by palmitate [12, 54] and lactate [59–61] have been recently reported. At the same time, it has been shown that metabolic heterogeneity can arise in genetically homogeneous cells as simple as the budding yeast *Saccharomyces cerevisiae* [64]. scFBA has the potential to highlight possible metabolic heterogeneity also within a genetically homogeneous population of cancer cells. The validation of the predicted interactions requires however non-trivial *ad hoc* experiments. As current techniques do not allow for easy determination of metabolites at the single-cell level, the heterogeneous population of alive cells should be first sorted to separate it into the sub-populations identified by scFBA. However, to sort cells based on fluorescent labeling (Fluorescence activated cell sorting), further analyses are necessary to possibly identify markers differentially expressed by the sub-populations. Less direct approaches might be taken, for example, by measuring the growth rate of wild-type cell populations and mutant populations in which the cooperation has been prevented (e.g. by blocking secretion or uptake of involved metabolites) and comparing with the model predictions. At this purpose, it should be assessed whether the metabolic interactions identified by scFBA are actually advantageous for tumor growth or are just related to the entropy of the system, i.e., to the fact that a configuration in which interactions among heterogeneous phenotypes take place is more likely than a configuration of identical and independent phenotypes. We address the issue in the following paragraphs.

#### Effect of cooperation on growth

The optimal values for the cooperation fluxes reported in Fig 4 display a larger variability than the optimal growth rates (Fig 2), even though much lower as compared to popFBA settings (by at least 60%). Therefore, we verified that the interaction among cells, given their transcriptomes, improves the capability of the overall population to achieve metabolic growth, while also correcting for the possible presence of thermodynamically infeasible loops [65]. At this aim, we compared the population growth rate of the case in which cooperation reactions are allowed, with the case in which they are not, given the same constraints (type 1 and type 2). As the mere secretion of metabolites (such as lactate) in the external environment (e.g., the blood) can improve growth rate, under given boundary conditions (e.g., limiting oxygen [4]), in order to allow for a meaningful comparison, in our experimental setting metabolites that can be secreted in the TME can also be secreted directly in the blood supply. By doing so, when the cooperation reaction is removed, the cell can still rid off of excess metabolites, which cannot however be taken up by other cells.

Remarkably, we observed that the ratio of the total biomass obtained in the absence of cooperation reactions over that in their presence may be lower than 1, implying that removal of cooperation limits the capability to achieve growth. In particular, we observed a ratio of: 0.90 for the LCPT45 dataset; 0.99 (H358); 0.99 (LCMBT15); 0.76 (BC04); 0.95 (BC03LN).

Intriguingly, but not surprisingly, the impact of cooperation prevention is higher on those datasets corresponding to more heterogeneous populations (LCPT45 and BC04). Intuitively, cells specialized in different metabolic programs are more likely to benefit from cooperation, as compared to similar cells.

#### Effect of cooperation on ATP production

For the sake of simplicity, in this study we assumed an optimal growth rate for the overall population, yet other assumptions may be readily investigated with the scFBA approach. Among others, it is common practice in constraint-based modeling to optimize for ATP production [66, 67]. As a proof of principle, we repeated the analysis on the effect of cooperation when the objective function is the total ATP produced by the population. We obtained the following ratios for the 5 datasets: 0.77 (LCPT45); 0.97 (H358); 0.93 (LCMBT15); 0.99 (BC04); 0.87 (BC03RLN). The observed discrepancies in the extent of the effects of cooperation inhibition on growth and energy productions are worth of interest and would deserve further investigation. Notably, both the energy production and growth rates of the H358 (cell line) population, which is expected to be homogeneous, are not affected by cooperation prevention.

### Boundary conditions affect scFBA predictions

Both in popFBA and in scFBA the cells were able to collaborate metabolically. As the integration of scRNA-seq data greatly reduced the space of feasible FBA solutions, those data encode information on how nutrient utilization should be distributed amongst the individual cells. Some cells that can no longer carry out a certain part of a pathway let their neighbors do this. However, it should still matter which nutrients are available to all cells. For a deeper characterization of given cancer populations, exo-metabolomic measurements to constrain the population boundary conditions would thus be needed. An exhaustive sensitivity analysis of scFBA results to boundary conditions is out of the scope of this work. However, it is interesting to compare the conditions in which the two major metabolites involved in cooperation (i.e., lactate and palmitate) are externally supplied to the population or must be produced endogenously.

**Fig 5:**
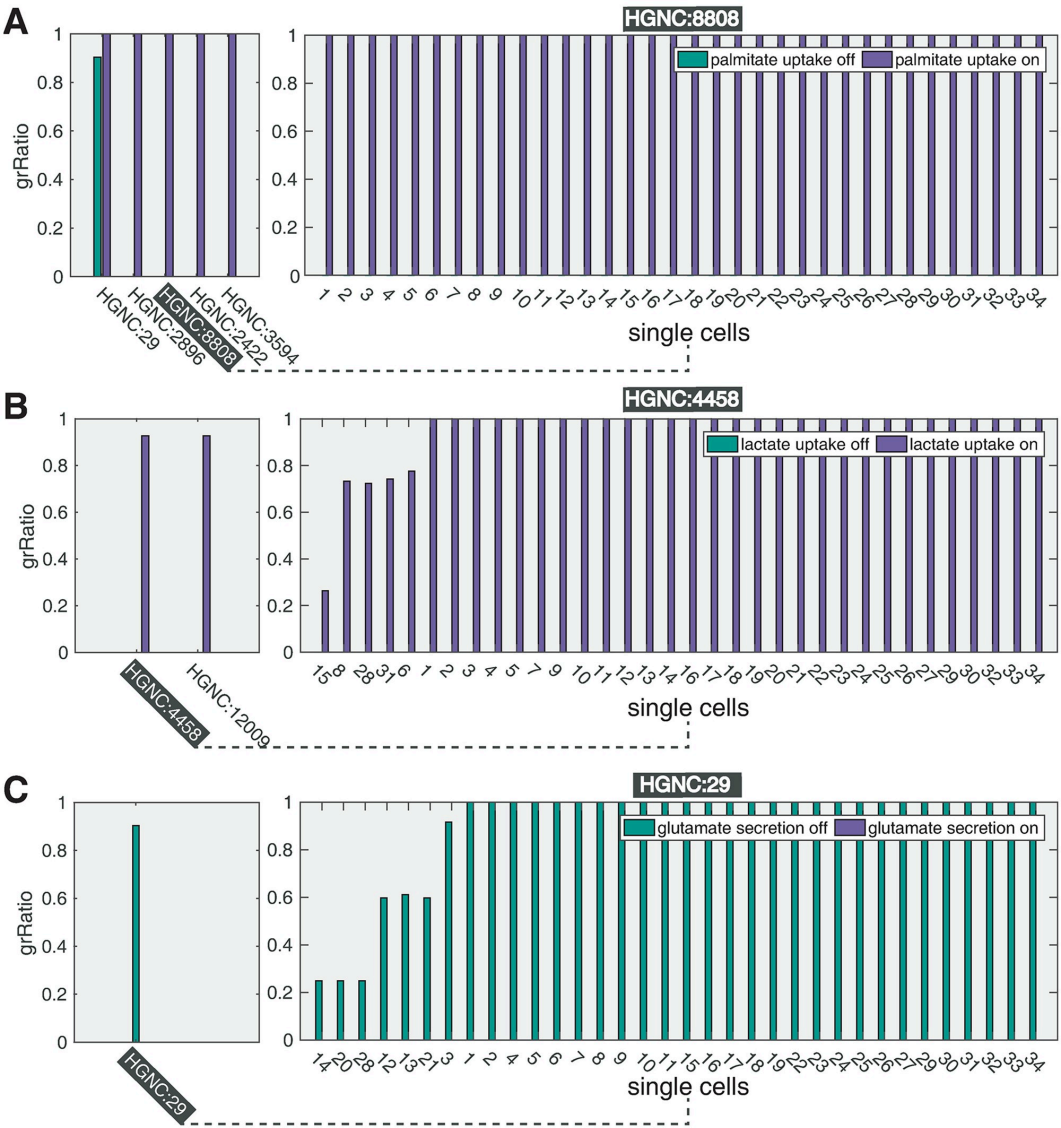
Impact of boundary conditions on gene-deletion predictions for LCPT45 dataset. A) Left: effect of gene deletions on the population growth rate, when exogenous palmitate uptake is allowed (purple bars) and when is not (green bars). Only genes with differential effect are reported. A missing bar indicate an essential gene (*grRatio* = 0). Right: effect of the deletion of gene HGNC:8808 on the growth rates of each single-cell. B) Left: effect of gene deletions on the population growth rate when exogenous lactate uptake is allowed (purple) and when is not (green). Right: effect of the deletion of gene HGNC:4458 on each single-cell. C) Left: effect of gene deletions on the population growth rate when endogenous glutamate release is allowed (purple) and when is not (green). Right: effect of the deletion of gene HGNC:29 on each single-cell.

Notably, we observed that uptake of exogenous palmitate does not affect the biomass production rate, indicating that no growth advantage is conferred by free availability of lipids. This result is in line with experimental evidence that cancer cells rely on *de novo* synthesis of palmitate-derived lipids [3]. However, in the baseline setting (no external palmitate supplied), we observed a group of cells that uptake the palmitate secreted by others (Fig 4). We verified that, once internalized in those cells, palmitate is not processed by the beta-oxidation pathway, but directly contributes to the biomass synthesis, supporting the evidence reported in [68], that an exogenous source of fatty acids can substitute for de novo synthesis in promoting cell proliferation and attenuate the cancer-specific toxic effect of lipogenesis inhibitors. It has also recently been pointed out that a limited access to environmental lipids may render the cancer cells more sensitive to the inhibitors of lipogenesis [68]. In line with these findings, it can be observed, with regard to the LCPT45 population (Fig 5A), that a set of genes stops being essential when exogenous palmitate is supplied. As expected, this set mainly includes genes directly involved in the synthesis of palmitate, namely: citrate synthase, fatty acid synthase and pyruvate dehydrogenase. The expansion of the plot in Fig 5A (left) shows that the latter (pyruvate dehydrogenase, ID: HGNC:8808) is essential for each cell within the population. It should indeed be noted that when the synthesis of palmitate is prevented in all cells, and exogenous palmitate is not supplied, also cells that used to rely on the palmitate synthesized by other cells must be affected.

As opposed to palmitate, the metabolite lactate is not strictly required for growth. However, it can be observed in Fig 5B that the deletions of genes encoding for glucose-6-phosphate isomerase (HGNC: 4458) and for triosephosphate isomerase 1 (HGNC: 12009)—two important steps for the utilization of glucose through glycolysis are not essential when lactate uptake is allowed, suggesting that lactate may be able to replace glucose as carbon source. Interestingly, when lactate uptake is prevented, the plot expansion in Fig 5B (left) shows that the gene HGNC: 4458 is essential in many but not all cells.

Also the set of metabolites allowed to be released, e.g., in the blood may affect the effect of gene deletions. For instance, if glutamate secretion is prevented, the deletion of the gene that encodes palmitate secretion becomes essential, as shown in Fig 5C. Remarkably, it has been reported that secretion of lipids facilitates tumour progression [69], whereas inhibitors of glutamate release have been proposed as new targets for breast cancer-induced bone-pain [55]. scFBA may enable to shed light on how the disposal of carbons through these two metabolites relates with the utilization pattern of exogenous nutrients.

## Discussion

We have here introduced scFBA to solve the problem of reconstructing the potential single-cell fluxome, starting from single-cell transcriptomes, by taking into account environmental constraints, as well as cell-cell interactions. Importantly, scFBA is able to point out the metabolic interactions that are established within a cell population.

scFBA integrates sc-transcriptomics data with (bulk) extracellular fluxes of the same cancer cell population, by means of a computational approach inspired to complex systems science [70]. A limitation of our approach is that it uses mRNA levels as a proxy of the maximal velocity of reactions (*Vmax*), thus neglecting the many factors that contribute to determine the expression level of a protein [71], as well as the role played by binding affinities of proteins in determining the *Vmax*. However scFBA does not predict the single-cell fluxome as a linear function of the assumed Vmax: the constraints on mass-balance and on the availability of substrates, as determined by the rates of consumption/secretion of nutrients by the entire population and by the requirement for the tumour mass to grow, are simultaneously taken into account. scFBA might also be implemented by using sc-proteomics rather than transcriptomics, should the former become available.

Although we do not explicitly model spatial organization, the constraints on single-cell transcriptome should implicitly preserve the information on the usage/secretion of most nutrients of each cell in its original position. The method seems also to neglect communication between cells through growth factors. In reality, it mostly does not if the growth factors act by changing transcription. But if they act by phosphorylating enzymes, then this is not taken into account. In its current form scFBA can however already be implemented to investigate why metabolic drugs are often ineffective and to provide indications for more effective treatment.

As a proof of principle, we have successfully applied the methodology to LUAD and breast cancer datasets. We have shown that the integration of scRNA-seq greatly reduces the space of feasible solutions that sustain metabolic growth of the overall population, which is prerequisite of tumour growth. This reduction allowed us to restrict the set of candidate drug targets, by eliminating targets that may seem obvious for the bulk, but do not work for heterogeneous populations, and, on the other hand, by revealing targets whose relevance can be appreciated only if cooperation among heterogeneous cells is accounted for. We have also illustrated that scFBA is valuable to extract features (i.e., the sc-flux values) from scRNA-seq data, in order to identify metabolic clusters of cells, which may be used to investigate other fingerprints of the cancer metabolic deregulation.

Although popFBA assumes that the cells achieve optimal growth, this assumption is mitigated in scFBA, by taking into account the transcriptional constraints. Moreover, we have shown how alternative objective functions, such as ATP maximization, may be investigated. Sub-optimality may also be taken into account by using sampling methods [72].

In this study, we have used scRNA-seq obtained with protocols based on C1 Single-Cell Auto Prep System, which have the advantage of allowing to remove dead cells before sequencing but may suffer from including low numbers of cells per sample (34—55 cells), as compared to modern emerging technologies which allow to obtain the single-cell transcriptome of thousands of cells at the cost of a bulk experiment [73], with an improved number of genes/transcripts per cell. In the future, our approach may be readily generalized to this kind of data. As illustrated in S4 Fig, the time of a scFBA computation increases linearly with the number of simulated cells and with the size of the template metabolic network. Alternatively, bootstrap-like methods can be adopted to reduce the number of cells considered in a single computation, while parallelizing the simulation of many smaller systems.

A major challenge, when dealing with scRNA-seq, is the presence of false-negatives. As a first approximation, we have used a bulk RNA-seq expression filter, where single-cell expression values of transcripts never detected in scRNA-seq but detected in bulk RNA-seq are replaced by the bulk values. For more reliable genes, we preserve information on cell heterogeneity, but we mitigate the risk of false-negatives setting the bound of the associated reactions to a small value *ϵ* rather than completely removing it. The choice of the value of *ϵ* is partially arbitrary. However, we verified that main results of our work are robust with respect to this choice (S1 Text and S1 Fig). The methodology might be refined, by combining it with more sophisticated data pre-preprocessing techniques, which may also take into account the specific quality parameters of the dataset.

Finally we have shown how constraints on the nutrient consumption and secretion rates (extracellular fluxes) of the specific sequenced population may affect scFBA predictions. As opposed to intracellular fluxes, (bulk) extracellular fluxes might be readily estimated, e.g., by approximation from metabolite concentrations in spent medium (exo-metabolome), when culturing patient-derived cells.

Hence, measurements of both extracellular fluxes and single-cell transcriptional information of the same heterogeneous cancer populations are needed to make scFBA predictions fully reliable. These datasets can be realistically obtained by culturing population of cancer cells and then processing the cells with scRNA-seq technologies and analyzing their spent medium with biochemical analyzers. Experiments under a controlled setting, e.g., a co-culture of metabolically characterized cancer cell lines, may first be performed to validate and tune the capability of scFBA to identify and measure the prevalence of different metabolic subpopulations of cells. The application of scFBA to analyze datasets more representative of tumor heterogeneity, obtained, for instance, by culturing cells from human biopsies, xenografts or organoids, will then pave the way to cancer personalized medicine.

## Supporting information

S1 Fig Sensitivity of scFBA results to *ϵ* for LCPT45 dataset. A) Left: histogram of biomass produced by each single cell when *ϵ* = 0. Right: Total biomass produced by the population of cells as a function of *ϵ*. The inset reports the same curve zoomed in on low *ϵ* values. B) Clustergram (distance metric: euclidean) of the effect of single gene deletions performed on scFBA for different values of *ϵ*, popFBA and bulkFBA. Growth ratio (grRatio) = 1 indicates totally redundant genes, while grRatio = 0 indicates lethal genes. C) Elbow analysis comparing cluster errors for *k* = 1, …, 20 (k-means clustering). Each curve refers to a different values of *ϵ*. D) Impact of cooperation among single cells for different values of *ϵ*. Curves refer to the ratio of total biomass (blue curve) and ATP (orange curve) produced by population models when cooperation reactions are blocked as compare to when they are allowed.
(TIF)

S2 Fig scFBA vs. popFBA. A) Dataset H358. Variability of the fraction of the biomass synthesis flux (logarithmic scale) for each cell over the population growth rate (left panel) before (purple) and after data integration (green). Effect of gene deletion (bars in right panel) on the population growth rate before (popFBA), after data integration (scFBA), and for the template metabolic network A* (bulkFBA). When *grRatio* = 0 (essential gene), the corresponding bar is not displayed. B-C) Same information as in A for LCMBT15 and BC03LN datasets.
(TIF)

S3 Fig Clustering of transcripts vs. fluxes. A) H358 dataset. Clustergram (distance metric: euclidean) of the transcripts of the metabolic genes included in metabolic network (left) and of the metabolic fluxes predicted by scFBA (middle). Right panel: elbow analysis comparing cluster errors for *k* ∈ {1, ⋯, 20} (k-means clustering) in both transcripts (blue) and fluxes (green). B-C) Same information as in A for the datasets LCMBT15 and BC03LN. D) Silhouette analysis for LCPT45 transcripts (left) and fluxes (right), when *k* = 3. Red dashed lines indicate the average silhouette for the entire dataset.
(TIF)

S4 Fig scFBA computation time. The linear relationship between the time for an FBA (and thus a scFBA) optimization and the size of the network is well established. We estimated the computation time required to perform a complete model reconstruction, from a template metabolic network to a population model with RASs integrated, for different number of cells (1, 10, 100, 1000 and 10000). We tested both our HMRcore metabolic network (panel A) and the genome-wide model Recon2.2 [51] (panel B). The former included 315 reactions and 256 metabolites, the latter is composed of 7785 reactions and 5324 metabolites. We were not able to reach the maximum population model size (10000 cells) with Recon2.2 due to insufficient RAM for 1000 cells. We also verified the feasibility of an FBA optimization for HMRcore and 10000 cells considered (2940021 reactions and 2350021 metabolites in total). The optimization required about 321 seconds. All tests were performed using a PC Intel Core i7-3770 CPU 3.40GHz 64-bit capable, with 32 GB of RAM DDR3 1600 MT/s.
(TIF)

S1 Text Description of sensitivity of scFBA results to *ϵ*. (PDF)

S2 Text Evaluation of clustering goodness. (PDF)

S1 Table Comparison of the fluxes of the two main clusters in Fig 3A-middle. (XLSX)

S2 Table Comparison of the fluxes predicted by scFBA, GIMME and iMAT with respect to LCPT45 dataset. (XLSX)
