# Supplementary figures and images for: Integration of single-cell RNA-seq data into population models to characterize cancer metabolism
Source: PLoS Comput Biol. 2019 Feb 28;15(2):e1006733. doi: 10.1371/journal.pcbi.1006733 (PMC6413955; doi:10.1371/journal.pcbi.1006733)

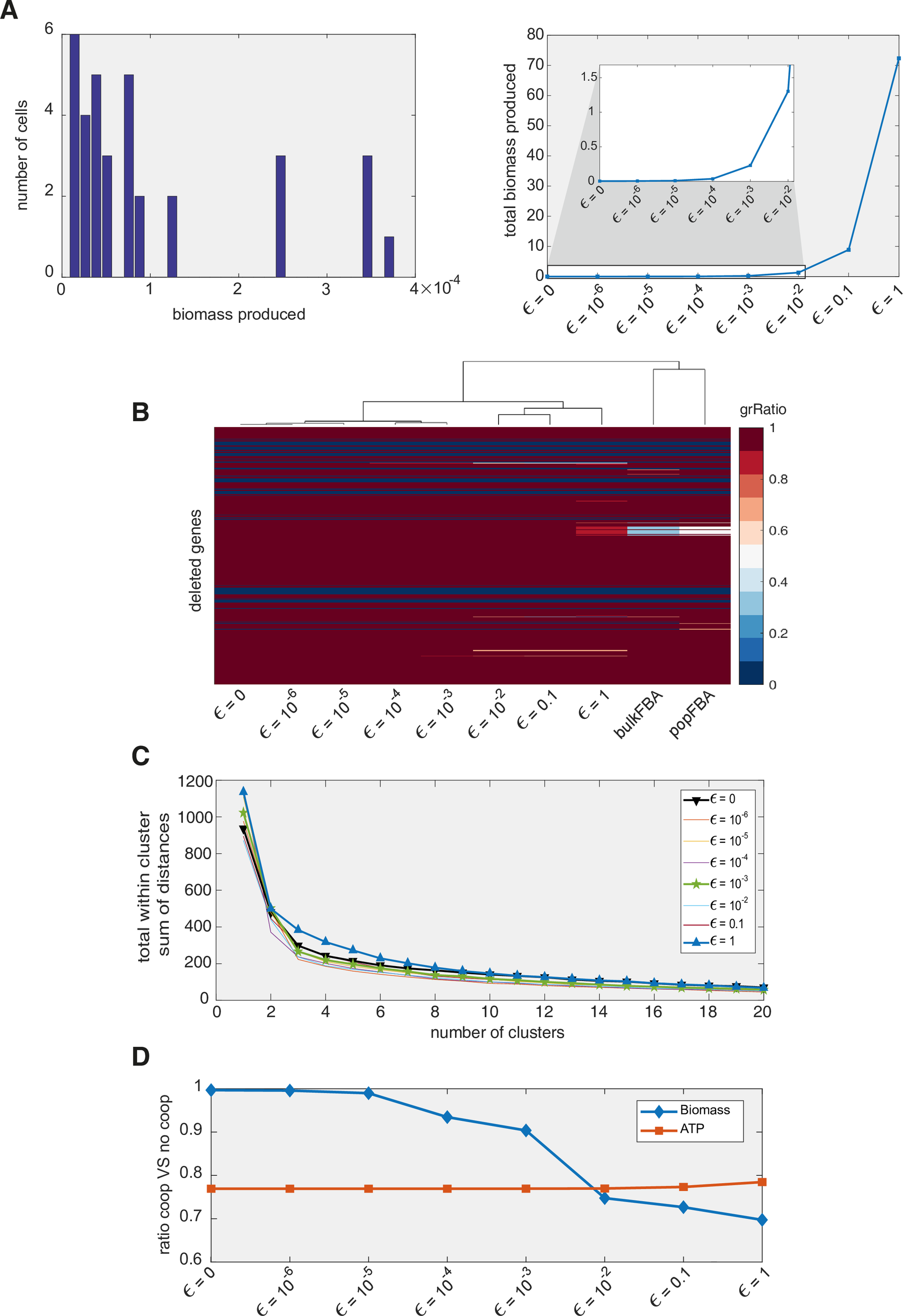

Supplement: S1 Fig — A) Left: histogram of biomass produced by each single cell when ϵ = 0. Right: Total biomass produced by the population of cells as a function of ϵ. The inset reports the same curve zoomed in on low ϵ values. B) Clustergram (distance metric: euclidean) of the effect of single gene deletions performed on scFBA for different values of ϵ, popFBA and bulkFBA. Growth ratio (grRatio) = 1 indicates totally redundant genes, while grRatio = 0 indicates lethal genes. C) Elbow analysis comparing cluster errors for k = 1, …, 20 (k-means clustering). Each curve refers to a different values of ϵ. D) Impact of cooperation among single cells for different values of ϵ. Curves refer to the ratio of total biomass (blue curve) and ATP (orange curve) produced by population models when cooperation reactions are blocked as compare to when they are allowed. (TIF) [file pcbi.1006733.s001.tif]

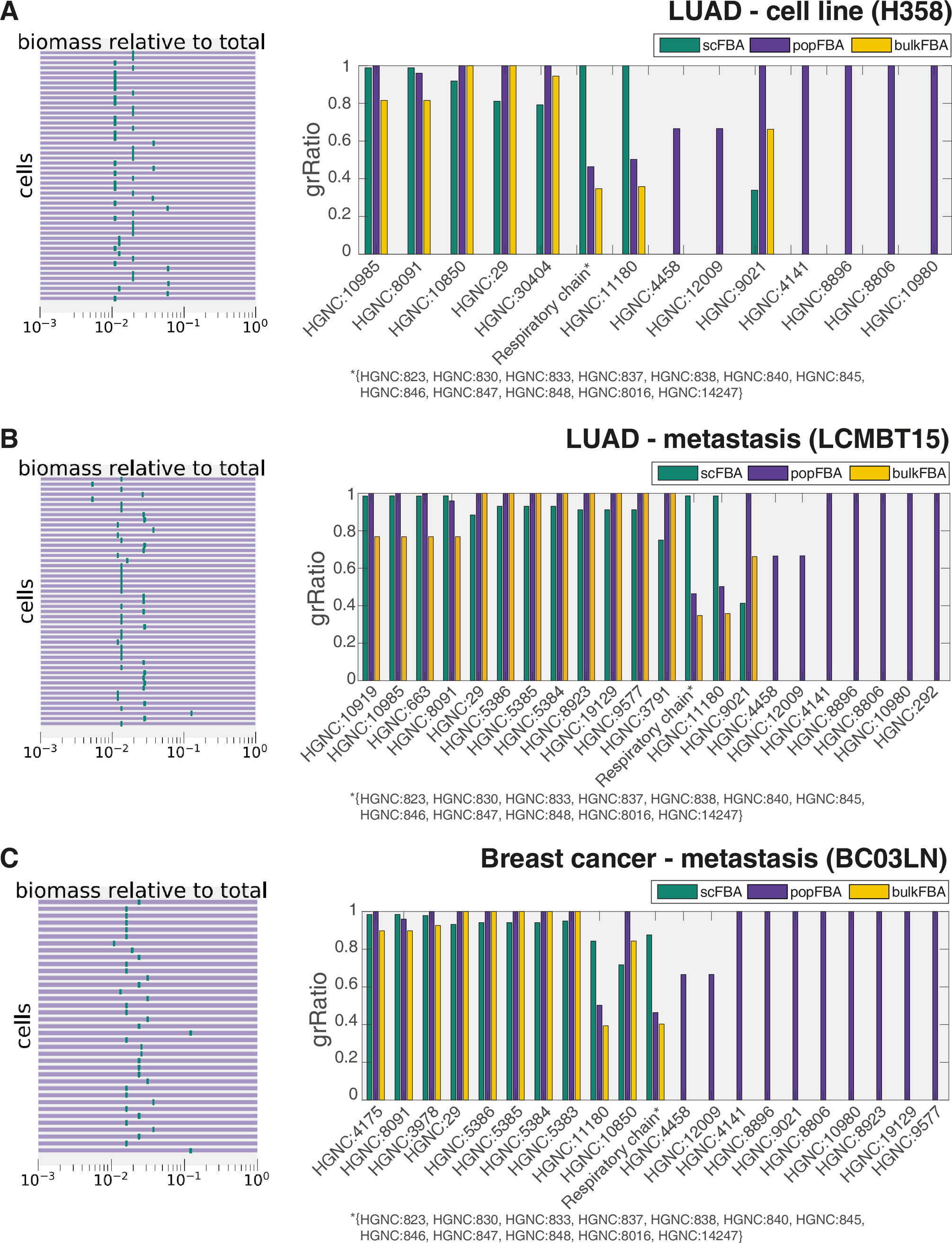

Supplement: S2 Fig — A) Dataset H358. Variability of the fraction of the biomass synthesis flux (logarithmic scale) for each cell over the population growth rate (left panel) before (purple) and after data integration (green). Effect of gene deletion (bars in right panel) on the population growth rate before (popFBA), after data integration (scFBA), and for the template metabolic network A* (bulkFBA). When grRatio = 0 (essential gene), the corresponding bar is not displayed. B-C) Same information as in A for LCMBT15 and BC03LN datasets. (TIF) [file pcbi.1006733.s002.tif]

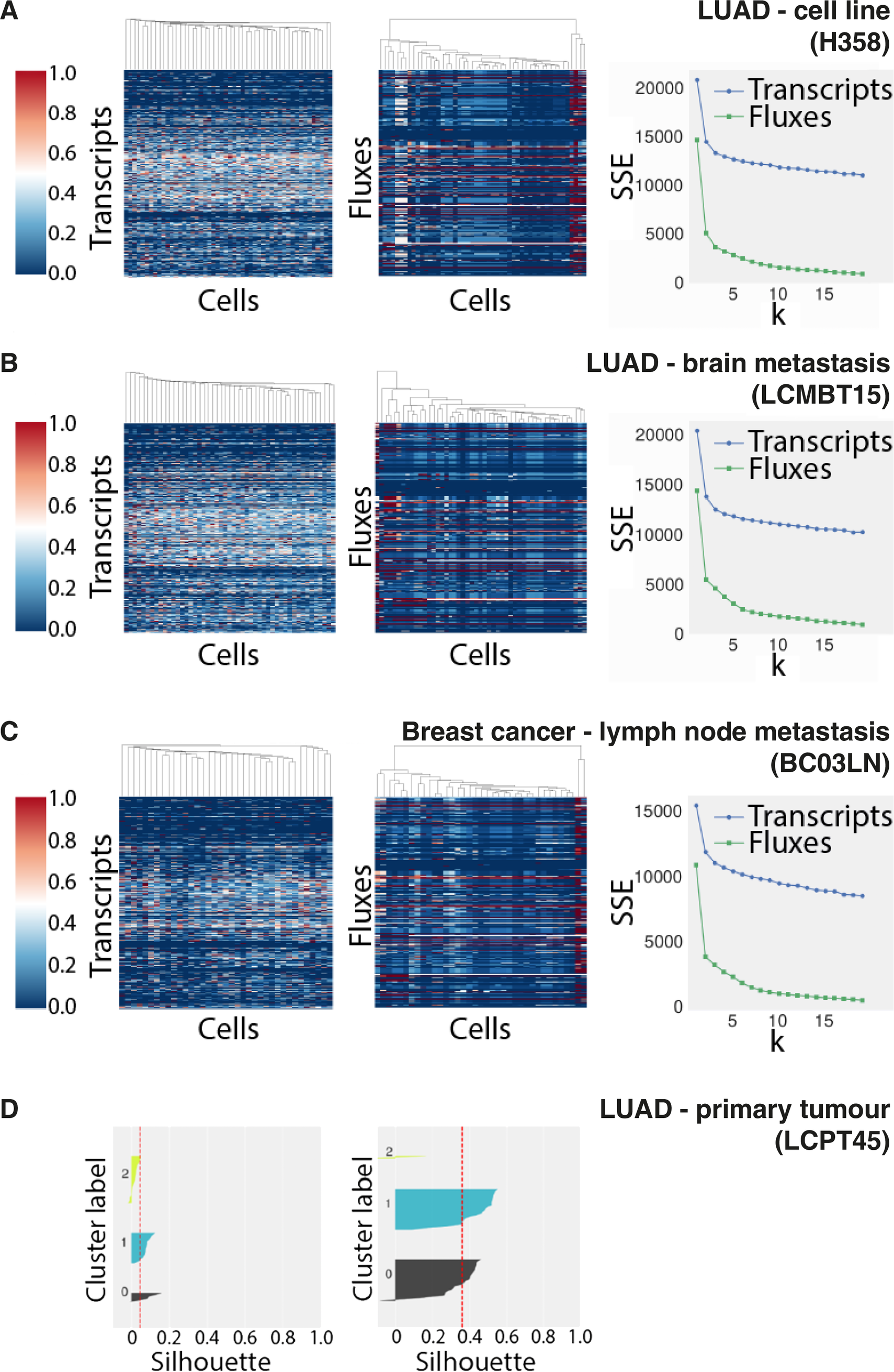

Supplement: S3 Fig — A) H358 dataset. Clustergram (distance metric: euclidean) of the transcripts of the metabolic genes included in metabolic network (left) and of the metabolic fluxes predicted by scFBA (middle). Right panel: elbow analysis comparing cluster errors for k ∈ {1, ⋯, 20} (k-means clustering) in both transcripts (blue) and fluxes (green). B-C) Same information as in A for the datasets LCMBT15 and BC03LN. D) Silhouette analysis for LCPT45 transcripts (left) and fluxes (right), when k = 3. Red dashed lines indicate the average silhouette for the entire dataset. (TIF) [file pcbi.1006733.s003.tif]

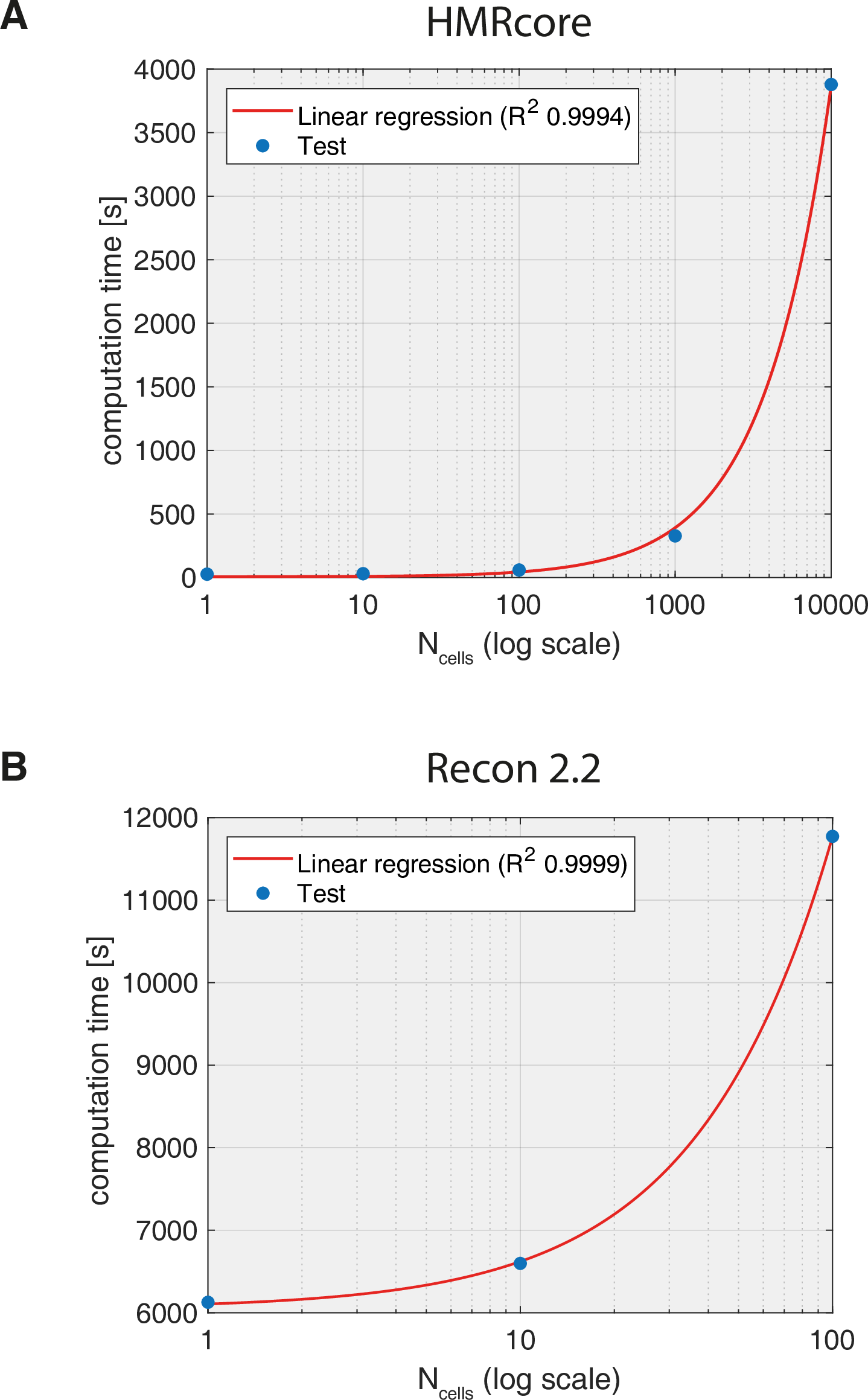

Supplement: S4 Fig — The linear relationship between the time for an FBA (and thus a scFBA) optimization and the size of the network is well established. We estimated the computation time required to perform a complete model reconstruction, from a template metabolic network to a population model with RASs integrated, for different number of cells (1, 10, 100, 1000 and 10000). We tested both our HMRcore metabolic network (panel A) and the genome-wide model Recon2.2 [51] (panel B). The former included 315 reactions and 256 metabolites, the latter is composed of 7785 reactions and 5324 metabolites. We were not able to reach the maximum population model size (10000 cells) with Recon2.2 due to insufficient RAM for 1000 cells. We also verified the feasibility of an FBA optimization for HMRcore and 10000 cells considered (2940021 reactions and 2350021 metabolites in total). The optimization required about 321 seconds. All tests were performed using a PC Intel Core i7-3770 CPU 3.40GHz 64-bit capable, with 32 GB of RAM DDR3 1600 MT/s. (TIF) [file pcbi.1006733.s004.tif]
