# Supplementary material for: Integration of single-cell RNA-seq data into population models to characterize cancer metabolism
Source: PLoS Comput Biol. 2019 Feb 28;15(2):e1006733. doi: 10.1371/journal.pcbi.1006733 (PMC6413955; doi:10.1371/journal.pcbi.1006733)
Supplement: S1 Text — (PDF) [file pcbi.1006733.s005.pdf]

S1 text. Sensitivity or scFBA results to epsilon.

The choice of the value of epsilon is partially arbitrary. It is therefore important to show how robust results are with respect to this choice.

At this purpose, we focused on the LCPT45 dataset and varied epsilon in the range {0, 10^{-6}, 10^{-5}, 10^{-4}, 10^{-3}, 10^{-2},0.1, 1}. Note that the value 1 cannot be considered as small, as the constraints become so loose (closer to the maximum flux value obtained with FVA) that we are losing information on single-cell heterogeneity, however, we used it as term of comparison.

Figure S1 reports the variation of main scFBA predictions as a function of epsilon.

It can be observed that, although quantitative results can depend upon the choice of epsilon in a complex manner, the main conclusions of the work are not affected. In particular:

- When epsilon takes value 0, the scFBA problem is still feasible, but we obtain very small values for the fluxes. For instance, the biomass values of the single-cells are of the order of 10e-4 when epsilon takes value 0 (Figure S1A left). As expected, the biomass value increases linearly as a function of epsilon (Figure S1A right).
- The effect of gene deletions, as described by the growth rate ratio, is robust with respect to the choice of epsilon (clustergram in Figure S1B). For the sake of completeness, we also included in the clustergram the values obtained with the population model when all transcriptomics-derived constraints are removed (popFBA), and those obtained when the individual template metabolic network A* is simulated, retaining information on on-off reactions only (the set Goff is deleted). We refer to the latter simulation setting as bulkFBA. The obtained values are indeed very similar for low values of epsilon ({0, 10^{-6}, 10^{e-5}, 10^{e-4},10^{e-3},10^{e-2}}), less similar for higher values ({0.1,1}), very different when no constraints (popFBA) or bulk constraints only (bulkFBA) are used.
- The paper conclusion that fluxes better cluster than transcript is robust with respect to the choice of epsilon. The elbow trend is indeed similar for different values of epsilon (Figure S1C), indicating that the optimal number of clusters is k=3, exception made for the high value 1, for which it is more difficult to assess the elbow point.
- The result that the interaction among cells improves the capability of the overall population to achieve metabolic growth and energy production is confirmed for all tested values of epsilon. Indeed, it can be observed in figure S1D that the ratio of the total biomass and of total ATP obtained in the absence of cooperation reactions, over that obtained in their presence, is lower than 1 for all the tested values of epsilon. The variation of the ratios is however not linear.
