# Supplementary material for: Integration of single-cell RNA-seq data into population models to characterize cancer metabolism
Source: PLoS Comput Biol. 2019 Feb 28;15(2):e1006733. doi: 10.1371/journal.pcbi.1006733 (PMC6413955; doi:10.1371/journal.pcbi.1006733)
Supplement: S2 Text — (PDF) [file pcbi.1006733.s006.pdf]

S2 text. Evaluation of clustering goodness.

The elbow method evaluates the variance explained as a function of the number of clusters. It assumes that the ideal number of clusters is the smallest *k* able to provide a good separation of the data (i.e., adding a further cluster does not significantly decreases the sum of squared errors, SSE).

When plotting the "cluster errors" (i.e. SSE) versus *k*, (Figure 3 and S3, right column), for small values of *k* there is typically a rapid decrease of the SSE; however, at a certain point the marginal gain in explained variance drastically decreases (an elbow in the plot).

The ideal number of clusters is identified at the elbow. For instance, in the right column of Figure 3 it is possible to notice that, for the fluxes relative to the primary tumour datasets, a sharper elbow is observed at $k=3$, hence the optimal number of clusters is 3, which corresponds indeed to the *k* identified by the hierarchical clustering analysis for these two datasets.

The silhouette score estimates for each element the cohesion (similarity of an object with other elements of its own cluster) and the separation indexes (dissimilarity with elements of other clusters). The silhouette has values in [-1,1]: scores close to 1 indicate that an element is correctly clustered, whereas scores close to -1 indicate a wrong assignment. A silhouette value around 0 indicates that the element is located among two different clusters.
